# Privileged but not in Power: How Asian American Tech Workers use Racial Strategies to Deflect and Confront Race and Racism

**DOI:** 10.1007/s11133-022-09527-1

**Published:** 2023-01-10

**Authors:** Tiffany Y. Chow

**Affiliations:** UC Santa Barbara, Santa Barbara, CA USA

**Keywords:** Asian Americans, Tech workers, Organizational inequality, Race

## Abstract

Research on tech workers has often focused on racial inequalities within the industry but has failed to seriously consider Asian American professionals as racialized subjects. This paper addresses this knowledge gap by centering Asian Americans as workers whose racial identity impacts their career trajectory and professional experiences in the high-tech industry. Based on 57 interviews with Asian American tech professionals, I find that Asian Americans use four main racial strategies to deflect or confront racism in the workplace Three of these racial strategies—racial maneuvering, essentializing, distancing— intentionally remove Asian Americans from the glare of racism. The fourth racial strategy, dissenting, acknowledges racism; workers using this racial strategy are often so frustrated by the white power structure of the high-tech industry that they find no other choice but to leave mainstream organizations. Several of these racial strategies are reinforced by local racial politics and the historical influence of Asian immigrant workers that helped shape both Silicon Valley and Asian American culture in the San Francisco Bay Area.

## Introduction

Research on Asian American professionals has often examined the perplexing issue of the racialized glass ceiling, colloquially referred to as the “bamboo ceiling.” Despite high socioeconomic and academic achievement, Asian Americans are often seen as unfit for leadership roles, assumed to possess personality traits better suited to being individual contributors than people managers and business executives (see: Berdahl and Min 2012; Chin 2020; Cheng 1996; Tinkler et al. 2019; Woo 2000). The repercussions for these prescriptive stereotypes can be felt even across Asian-concentrated occupations in the high-tech industry, where Asian Americans are the least likely racial group to become executive leaders in high-tech organizations (Gee and Peck 2018), suffer from “personality” penalties during the hiring process (Chavez 2020), and lack the social networks that would enable successful hiring or advancement in “mainstream” or white organizations (Choi et al. 2010; Petersen et al. 2000; Shih 2006). Nonetheless, Asian Americans are rarely centered in social science research as racialized subjects that encounter structural discrimination nor do Asian Americans themselves generally consider themselves as part of a discriminated group in the workplace (Chin 2020).

This article contributes to the current literature by identifying how Asian Americans deflect racial discrimination in a white-dominated high-tech industry. Because previous research has established that Asian Americans often find it difficult to acknowledge structural racism (Chin 2020; Chou and Feagin 2008; Tuan 1998; Woo 2000), I pay particular attention to how Asian American tech workers explain racialized interactions with colleagues or artificial barriers in their career trajectory. I analyze participant responses using the concept of a “racial strategy.” I have adapted this term from “gender strategies,” used in Chen’s (1999) analysis of Chinese American men and their masculinities, which was inspired from Hochschild (Hochschild and Machung 2012). I use the term racial strategies to refer to how Asian American tech workers “solve” the problem of race in a supposedly meritocratic industry.

I argue that strategies used to justify Asian Americans’ engagement or disengagement from racial politics at work reflect a variety of lived experiences, including local racial histories and exposure to cultural stereotypes. Racial stereotypes of Asian Americans, particularly around their supposed intellectual prowess and lack of social skills, shape Asian Americans’ understanding of their racial identities in a professional environment. Because current stereotypes about Asian Americans seem overwhelmingly positive, they often reinforce the idea that Asian Americans do not face racial discrimination, even given well-known barriers such as the “bamboo ceiling.”

Asian Americans’ understandings of racial stereotypes and their experiences with anti-Asian discrimination depends on their immigrant generation. Because native-born and immigrant Asians can face different forms of discrimination in the workforce and have different cultural understandings around stereotypes, this paper focuses specifically on the experiences and racial strategies of native-born and socially Americanized Asian Americans in the high-tech workforce. To explore the possible influence of regional cultures and demographic contexts on racial strategies and understandings, I conducted interviews in two labor markets with large tech hubs: Austin, Texas and the San Francisco Bay Area.

Drawing on 57 in-depth interviews, I find that where Asian Americans sit on a spectrum of racial inequality awareness informs the racial strategies they use to interpret anti-Asian discrimination in the tech labor market. Four racial strategies can be identified: racial maneuvering, essentializing, distancing, and dissenting. On one end of the spectrum, racial maneuvering is the refusal to engage with the possibility of anti-Asian racism; on the other end, dissenting is the acknowledgement that one’s own professional career has been affected by anti-Asian racism. Two of the strategies, essentializing and racial maneuvering, rely on the internalization of racial stereotypes as a foundation for rejecting the possibility of anti-Asian racism. The other two strategies, distancing and dissenting, recognize the existence of anti-Asian racism but differ in whether subjects acknowledge its impact on their own professional lives.

Distancing as a racial strategy is particularly nuanced and sits at the cusp of racial consciousness for Asian American tech workers: while distancers accept that anti-Asian racism occurs professionally, they do not perceive it as an immediate concern and minimize its effects in their own professional lives. Unfortunately, many of those who used the dissenting strategy found that they needed to leave “mainstream” or white-dominated tech companies in order to gain control of their career trajectories. Asian Americans who grew up in majority-minority cities in the Bay Area were especially adept at de-racializing their professional lives, perhaps because of the role Asian Americans played in the development of Silicon Valley and their mainstream cultural acceptance in the region.

The study of a seemingly successful out-group, Asian Americans, can be instructive in understanding how coping strategies can be harmful and maintain workplace inequalities. I discuss the implications of racial dissociation by Asian Americans and how this may have reverberating consequences beyond Silicon Valley.

## Why Geography Matters for Asian Americans in High-Tech Organizations

The development of Silicon Valley is inextricably linked with Asian migration to the region, made possible after the passage of the landmark Immigration and Nationality Act of 1965, which abolished immigration quotas by nation (Lung-Aman 2015). Asian workers immigrated to California and took on a variety of low-skilled and high-skilled work, from assembly jobs to venture capitalism, and became key players in the transformation of the local economy from agriculture to high-tech (Li and Park 2006; Lung-Aman 2015; Saxenian 2006). The influx of Asian workers also altered the racial and cultural landscapes of Bay Area suburbs from white-majority to majority-minority (Jiménez 2017; Li and Park 2006; Lung-Amam 2015). The impact of a rapidly expanding Asian workforce was reflected in the social geography of the region, where Asian malls, residencies, and cultural institutions established Asian communities as part of the region’s economic and social fabric (Lung-Aman 2015).

That is, Asian Americans were “place-makers” in Silicon Valley suburbs and have actively worked to make their neighborhoods reflective of their needs and desires (Lung-Aman 2015). The concentration of Asian Americans in California, coupled with an active investment into suburban life, has created an environment where various Asian ethnic cultures are publicly visible, celebrated, and considered part of the cultural mainstream (Jiménez 2017; Tuan 1998). As a result, Asian Americans in California often possess racial privilege, defined here as a privilege where Asian Americans do not have to actively consider their racial identity in their day-to-day lives, a privilege generally associated with whites (Gans 1979; Tuan 1998; Waters 1990).

This particular form of racial privilege enables California-based Asian Americans the option to disengage with race. On the individual level, being Asian is not necessarily a salient identity marker because of wide social acceptance. As one Bay Area product manager remarked, it was *white* classmates who were considered “token” friends and that they were aware and respectful of Asian cultural practices, even in public spaces such as the high school cafeteria, where Asian American students used chopsticks and brought ethnic food for lunch. Conversely, research has suggested that Asian Americans who grow up in majority-white regions struggle to fit in and face racial harassment (Tuan 1998). This population of Asian Americans has largely remained understudied in research on tech organizations because they are more likely to live outside of the Bay Area. Although this ability to disengage with race does not *only* happen with California Asian Americans, it is one way for Asian Americans to have an “optional” identity. Another way, as this paper explores, is when Asian Americans are situated in professions or job functions where they are surrounded by Asian peers, such as software engineering. I explore how being surrounded by Asian peers has the effect of “neutralizing” racial identity as an inequality factor in the findings.

Most studies about race and the high-tech industry have been situated in Silicon Valley without acknowledging the unique demographics of the region (see: Alfrey and Twine 2017; Chavez 2020; Shih 2006). In order to address this knowledge gap and to contextualize the racial frameworks of tech workers, I recruited subjects across the Bay Area as well as in Austin, Texas, where Asian Americans have a smaller and newer presence, growing from 3% of the overall population in 1990 to over 7% in 2016 (Robinson 2011, 2016). Broadening the recruitment pool from one geographic location to two provides a way to explore the fluidity of Asian American occupational experiences and consequently, expands the discussion on race in the high-tech industry.

## Asian Americans as Racialized Workers

In the post-war era, racial stereotypes about Asian Americans generally transformed in a positive direction, from the deviant, threatening and hypermasculine “Yellow Peril” to the hard-working, law-abiding and effeminate “Model Minority” (Chou and Feagin 2008; Hsu 2015; Okamoto 2014; Shek 2007). Although the Model Minority is a “positive” stereotype on the surface, it has been wielded by whites as a divisive weapon against other racial minorities and has been used to dismiss the notion that racism toward Asian Americans is still prevalent (Cheng 1997; Chou and Feagin 2008).

Furthermore, Deborah Woo explains that Asian Americans are not a model for *whites* but for *other* racial minorities: when Asians increase their presence in spaces previously reserved for whites, they are perceived as economic threats to white workers (Espiritu 2008; Tang 1993) and as competition for resources (Berdahl and Min 2012; Maddux et al. 2008). Studies show that even positive stereotypes of Asian Americans such as being intelligent and hardworking can engender negative emotions like hostility and jealousy. Individuals who perceive Asian Americans as highly competent may also feel threatened by them (Ho and Jackson 2006; Maddux et al. 2008).

Although research on Silicon Valley has generally focused on white tech workers, more recent analysis has taken an intersectional approach to consider the experiences of racial minorities and immigrant workers. Still, even when Asian tech workers are included in studies on minority experiences, the plurality of Asian identities and experiences are often glazed over: Asian nationals and native-born Asian Americans are often collapsed into one categorical group, and serious inquiry into the Asian worker experience using racial theory to frame the ways they both internalize and resist racial oppression are often left out of the analysis.

This paper offers a new perspective on Asian American tech workers by considering how a specific subgroup of native born or socially Americanized Asian workers interpret and internalize their racialization within the high-tech industry. As previous research has shown, Asian Americans do not possess a collective history of racism and oftentimes do not know how to respond to or recognize racism (Chou and Feagin 2008; Tuan 1998). The strongest collective racial memory for this group is the Model Minority stereotype and its associated superficially positive attributes, which often makes it difficult for Asian Americans to see themselves as a discriminated group, even when they have racist encounters (Chin 2020; Chou and Feagin 2008; Woo 2000).

Instead, Asian Americans, as well as other racial groups, often find other explanations for these incidents. Asian Americans often turn to their own perceived behavioral deficiencies to justify a glass ceiling (Chin 2020; Woo 2000) while others rely on racist stereotypes and bias to explain job outcomes (Chavez 2020; Xin 1997). Even within social science literature where structural discrimination against Asian tech workers is consistently documented (see: Chavez 2021; Hossfield 2005; Shih 2006), Asian American still appear in organizational literature as a group whose minority status is debated and whose oppression is openly ranked as less severe than other groups, rather than recognizing the specificity of their racial discrimination (Alegria 2019; Alfrey and Twine 2017; Franklin 2021).

This study introduces a new lens to the research on minorities in the high-tech industry by centering Asian Americans as racial subjects and theorizing about their understanding of racial inequality in the workplace.

## Research Design and Methodology

Because perceptions of inequality depend on location within institutions (Cech and Blair-Loy 2010), Asian Americans can help illustrate how a successful out-group understands race and racism within a racialized institution such as the high-tech industry. This study is part of a larger study on Asian tech workers. For this project, I draw on interviews with 57 Asian Americans who were either currently working in Austin or Silicon Valley or had, within two years of the interview, worked in either locale.

My personal and professional contacts were instrumental in recruiting Asian American tech workers across the two states. Because I previously worked in tech and lived in both locations, friends and former colleagues connected me through their networks and I used a snowball sampling technique to further expand my interview pool. Unexpectedly, I also received help from Ellen Pao, the former CEO of Reddit, whose experiences with gender discrimination against her former employer the venture capital firm Kleiner Perkins are well known within Silicon Valley circles. When Pao retweeted my recruiting request for Asian American tech workers on Twitter, it ended up being shared more than 800 times on the platform and reached workers in the Bay Area outside of my immediate social network.

My interview request was framed as a study of Asian American tech workers and their experiences. Because the interview covers sensitive information, including racial identity, racism, and sexism within the workplace, privacy and anonymity were of the utmost importance. As a result, I only reference ethnicities in instances when they would not identify the participant.

Interviews were conducted between February 2019 and March 2021. I was able to conduct half of my Austin-based interviews in person before the COVID-19 pandemic started. For the remainder of the interviews, I interviewed participants using a video-based platform (e.g. Google Hangouts) unless otherwise requested. Interviews generally averaged a little over an hour long (~ 70 min); in four cases, I scheduled two interviews with the participant to accommodate their schedules.

For the purposes of this study, I defined Asian American status in two ways: individuals who were born in the United States; or, those who had moved to America before the age of 13 and are considered socially Americanized as part of the 1.5 generation (Chin 2020). Immigrant generation makes a difference when considering the impacts of race in the workplace as immigrant generation often confers generation-specific disadvantages in the labor market in areas such as unemployment, lower annual salaries, and management responsibilities (Kim and Zhao 2014). That is, discrimination can look different for native-born Asian Americans compared with their immigrant counterparts. For instance, whereas wages are often used as a barometer of labor market parity, it is likely to be of greater concern for Asian immigrant workers than second generation Asian Americans (Kim and Zhao 2014; Wang et al. 2017). Areas of disadvantage may be less obvious for the latter group, where more subtle —and harder to pinpoint—expressions of racism may dominate their experience. By separating Asian immigrant workers from their native-born Asian Americans in the discussion on Asian tech workers, this paper can more seriously consider the specific ways the native-born and socially Americanized Asian Americans respond to their specific experiences around discrimination and their culturally American understanding of race and racism.

All but six interview subjects were either born in the United States or moved to the United States by age 9 except in two cases where the participant’s family first moved to Canada before moving to the States. In the latter case, one of the participants moved to Texas by age 12 while the other moved to California by age 14. I make an age exception for the latter because the participant has no accent and would not be socially categorized as a “non-native” Asian American.

Interviews were semi-structured and started with basic introduction questions around where they grew up and descriptions of their childhood and home life, how they came to work in tech, and progressed into conversations around past and current employers. If participants did not organically bring up the topic of racial stereotypes of Asian Americans at work, I asked them if they knew of such stereotypes and started a line of questions around whether these stereotypes were advantageous or disadvantageous in the workplace.

Most studies on tech organizations focus on software engineers; I expanded my interview pool to include workers from a variety of technical roles: product designers, user experience researchers, product managers, project managers, program managers, and content designers. This allowed me to document and analyze a wider variety of occupational experiences. I recruited participants who worked “in-house” (for example, at a company like Google) or at design consultancies; the latter group helps other companies innovate, particularly within enterprise software and mobile app development. Many traditional management consulting companies such as McKinsey and Deloitte now have design consultancy arms that employ designers and software engineers. It is fairly common to job hop between the two environments and participants often had experiences in both. While this study’s main goal is not to delineate the differences between different occupational paths within tech, I provide insight where it is relevant in my findings.

I recorded and transcribed all interviews and coded in multiple cycles using an inductive approach, refining and regrouping coding categories to construct the major themes of this paper (Saldaña 2015). Following each interview, I wrote analytic memos to better document what I understood to be important themes at the time and to track how my understanding of participant conversations evolved over time (Luker 2010; Saldaña 2015).

## Racial Strategies of Asian American Tech Workers

A through line that makes it viable for Asian Americans to develop racial strategies relies on the relative weakness of the Asian American collective memory on racism and the pervasiveness of the Model Minority stereotype as *the* racial identity for Asian Americans (Cheng 1997; Chou and Feagin 2008).

Sarah, an Asian American in her 50 s who grew up in a small town on the West Coast, recalls how deeply being a Model Minority shaped her own childhood experience.

When I grew up, the Model Minority was on the cover of Time [Magazine]...And definitely my parents wanted me to be better than everyone else not because to show everyone else [but] because they knew I had to be...I could not break the law. I could not go smoke pot. I mean, I know Asian Americans do those things now but [laughs], I do think there was an actual fear underneath that. If I did not behave like a Model Minority the discrimination, the hatred would kick in.

The Model Minority figure from Sarah’s childhood loomed as a threat: stepping outside of those stereotypes meant that she would be a target of hateful behavior. For many participants, the Model Minority stereotype was the main framing device they had in explaining their career paths: being Asian became synonymous with both being *better* than other racial groups and instructional in acceptable behaviors and ambitions in the workplace. That is, embracing a racial identity as a Model Minority often guided participants toward particular racial strategies.

Chen’s (1999) analytical concept of “gender strategies” is inspired by Hochschild’s use of the term (Hochschild and Machung 2012), originally used to describe how husbands and wives’ gender ideologies informed their actions and emotions around household management. Chen’s particular use of “gender strategies” refers to how Chinese American men create a “plan of action” to resolve the issue of negative stereotypes against them (Chen 1999). Namely, Chen asks how Chinese American men “achieve” masculinity in a society that has deemed them effeminate and undesirable.

I build on Chen’s concept of a “racial strategy” to explore how Asian Americans “solve” for the problem of racial stereotypes (e.g. inability to lead) in an industry that favors an aggressive white male “nerd masculinity” (Cooper 2000; Ensmenger 2015). That is, how do Asian Americans, who are generally stereotyped as passive, hard workers make sense of their career opportunities? How do they explain the “bamboo ceiling” or the lack of Asian American executives and tech founders? This research examines how Asian Americans wrestle with race and racism in an industry that has declared itself a meritocracy.

Four racial strategies emerged from participant interviews. Although three of these strategies deny the salience of race in subjects’ professional lives, these strategies differ in their positions on the existence of Asian racial inequality in the workplace. A fourth strategy directly acknowledges anti-Asian racism and requires subjects to be fully aware of the ways in which racism shapes their professional experiences. The strategies are:

1. Racial maneuvering, which exploits Model Minority stereotypes to establish Asian Americans as desirable employees and therefore not at risk for discrimination;
2. Essentializing, which relies on cultural stereotypes around personality characteristics to explain the lack of Asians in leadership;
3. Distancing, where individuals acknowledge anti-Asian racism but claim that it does not personally affect them;
4. Dissenting, where individuals acknowledge that racism impacts them in structural ways and often attempt to remove themselves from unfavorable situations.

Participants often use multiple strategies to explain their observations about the racial makeup of tech companies and to explain their own career outcomes. Several of these racial strategies are attractive for certain subgroups of Asian Americans. For instance, distancing is a popular method for both Bay Area Asian Americans and Asian American software engineers because both groups experience some form of racial privilege in their personal and/or professional lives. This privilege normalizes the representation of Asian Americans within tech culture and is used to dismiss the effects of racism in Asian American tech workers’ daily lives.

Although most of these racial strategies successfully removed participants from seeing themselves as an oppressed racial group, I wanted to note that the participants in this study were transparent about their own limitations in understanding race and racism, willing to be challenged about their frameworks in understanding tech as a meritocracy and shared personal experiences about their work cultures and personal experiences of being Asian American that were both painful and joyful.

In short, this group’s experience highlights just how successfully racialized stereotypes have been internalized but also provides potential pathways to reject white power structures. Many of these participants are second generation Americans whose parents did not understand American racism or history; additionally, most participants lacked the historical context to understand their experiences as shaped by larger forces than their interpersonal struggles. It is within this particular set of circumstances that makes it possible for Asian Americans to see themselves as a successful racial group within tech and to minimize anti-Asian experiences.

### Racial Strategy: Racial Maneuvering

Racial maneuvering as a strategy exploits racial stereotypes that Asian Americans have internalized and taken at face value. Under this strategy, Asian Americans assign their racial difference from the dominant white culture as a positive and consider themselves more qualified than white workers and therefore not at risk for discrimination. Within the spectrum of racial inequality awareness, maneuverers tend to reject the idea of anti-Asian discrimination and reflect no awareness of their own racialization, making it possible for racial stereotypes to dominate their understanding of their Asian American racial identity.

While racial maneuvering establishes the Asian American identity as a positive and can potentially be seen as an act of resistance to the white power structure, I find that Asian Americans often rely on racialized stereotypes in order to “do difference” and establish power. Instead of deconstructing internalized notions of the Model Minority stereotype, Asian Americans interview subjects in this study leaned into it to establish professional competence and desirability. It is not accompanied by a desire to address larger power structures that question why these stereotypes are so popularly used, nor does it empower Asian Americans to lobby for meaningful change. Nevertheless, this approach establishes two key points: one, it supports the idea that Asian Americans are advantaged and attractive workers, and two, recuses Asian Americans from being an oppressed group. Racial maneuvering situates Asian Americans as a group that does not encounter racism in the tech workforce and suggests that race, as a discriminatory mechanism, is not applicable to the Asian American work experience.

In this section, I demonstrate how the Model Minority stereotypes bolsters Asian American confidence in their desirability as workers but also dampens their awareness of the harm it can cause. I also explore how racial maneuvering is particularly heightened by the intersection between Model Minority stereotypes and the local history of Silicon Valley, where Asian Americans are well-represented in academic pursuits and high-tech organizations.

Jack, an entrepreneur and software developer, shared why he was interested in participating in a study on Asian Americans in tech:

[What] I would love to read about is how Asian American culture…. influenced the culture of tech companies. And that seems like a really interesting question to me because of how much I imagine the culture of tech companies has influenced the rest of America and the entire world through its products.

For Jack, Asian Americans are core not only to the identity and culture of Silicon Valley but “the entire world.” To understand how Jack is able to center his cultural identity as *the* influence of Silicon Valley, and consequently the world, it is important to describe the social and cultural world Jack lives in. Born, raised, and educated in the Bay Area, Jack has only lived and worked in environments where Asian Americans are socially, professionally, and intellectually respected and visible.

Jack’s high school was typical of many Silicon Valley high schools in that the student population is mostly white and Asian. Asian students at his high school made up 40% of the school population but nearly 90% of the high school’s Advanced Placement classes and extracurriculars. The racial distribution of Asian students in AP classes Jack describes is not unusual in Silicon Valley, where academic achievement—and overachievement—is often racially encoded as “Asian” (Jiménez 2017).

As a result, Jack’s high school understanding was that Asians were simply *better* at school. For instance, Jack described his teenage surprise when he found out white high school students won the interscholastic Quiz Bowl competition:

I found it weird when the winners of the Quiz Bowl, nationwide, were schools in Virginia that were mostly white. My assumption, my completely unquestioned assumption [up until that point], which I don't think I ever said explicitly or maybe even thought explicitly, was just like, of course Asian Americans...are just smarter, or are better at school.

Jack’s shock that white students could compete and even dominate in a battle of the intellects was genuine, given his own high school experience where Asians were synonymous with high academic achievement. Prior to this incident, Jack simply accepted what he saw: when compared with whites, Asians were clearly more high-achieving. Although college and his professional experiences have helped Jack understand his rarefied status as the child of highly educated immigrant parents and how that has shaped him and his Asian American peers into academically minded students, it is difficult for him to shake his adolescent understanding of race, in part because he is still based in the Bay Area.

Jack characterizes himself as a *more desirable* job candidate given that he is both an Asian American male and an alumnus of a prestigious university. He goes so far as to state that if employers were to ignore his bachelor’s degree, he would remain a better job candidate:

I'm an Asian American male, I just feel like people's assumption is going to be I am more likely to be competent than if I were... a white male. Which, now that I say that out loud, that's pretty weird... I have zero evidence for that and I have zero certainty in that, really. But that's how I feel.

Jack’s professional framework about the desirability of Asian tech workers is informed by his fundamental understanding of what it means to be Asian American in general, which is *to be better* than his white peers. That is, whereas Deborah Woo describes the Model Minority as a model for other racial minorities, Jack’s understanding of Asian ability is in direct comparison with white peers (Woo 2000). Jack’s core beliefs about white-Asian racial dynamics are so ingrained that he projects that *others* (“I just feel that people’s assumptions…”) have the same views about Asian technical competency.

Although Jack in many ways demonstrates an extreme form of racial maneuvering, where his professional framework goes so far as to center Asian Americans as the central figures of tech culture, other participants also relied on stereotypes to racially maneuver Asian Americans into desirable tech workers. For instance, Asian American participants regularly shared that they have received benefits during the job interview process. Ana, an Austin-based designer, shared that when she was looking for a job, her hiring manager, who was also Asian, made an offhand remark about Ana being a “safe bet” because she was Asian and went to a highly selective East Coast university. Although Ana shared that she found the interviewer’s notes flattering in the moment, she also shared that it was “unfair [for him] to make that judgment… if anything, that situation put me ahead because I was Asian American.”

Situations in which Asian Americans seem to benefit unfairly in the labor market made it difficult for them to call out other instances in which they were more negatively racialized. Ana, for example, mentioned that she had been repeatedly called by another Asian American colleague’s name, but nevertheless felt her experience in tech was similar to white peers and never felt inhibited in her career opportunities.

Other participants pointed out that racial stereotypes advantaged Asian Americans in the engineering field and took these “wins” at face value. Bo, an Austin-based software engineer noted that Asian Americans are “stereotypically the smart kids… we’re great at math and obviously not all of that is true, [but] it’s the perception.” From his perspective, Asian Americans “haven’t been discriminated against [in terms of] opportunities to become software developers or working in the high-tech industry, period.” In particular, Bo makes a distinction between “low level” and “historic” discrimination, where Asians may face interpersonal (“low level”) discrimination in moving up the corporate ladder but otherwise have benefitted from positive stereotyping that other racial minorities do not.

Bo touches on the common misconception that Asian Americans do not face structural (“historic”) discrimination. Like many participants, Bo is not aware of Asian American history nor is he able to connect his current experience to past racial trauma. Bo instead maneuvers Asian Americans outside the realm of structural discrimination and into a place of advantage. From his perspective, Asian Americans are an exception to the oppression faced by other racial minorities. Many individuals I interviewed stated that while they are people of color, their usage of that word does not “come with the social implications of other people of color.”

In other words: Asian Americans see themselves as removed from the everyday struggles of most racial minorities at work. A consequence of this framework, however, is that these so-called advantages minimize Asian American struggles in the tech workplace because they seemingly possess so many privileges. For instance, because Jack has had incredible upward mobility as a software engineer and as a company co-founder, he had a difficult time imagining why underrepresentation at the C-suite remains a critical issue:

I don’t feel a sense of dismay [about the bamboo ceiling], which I might expect myself to…. I’m aware of so many problems facing so many people and so many problems facing minorities, and so many problems facing Asian Americans who are not Chinese or Taiwanese or Indian Americans working in tech. I just have a hard time caring that much about, this is just my emotional reaction thinking through...[the fact that] Asian Americans are underrepresented at the C-suite level.

Jack’s real-time processing on why he lacks interest in the bamboo ceiling is honest and revealing: why should he care about other Asian American in tech, who are overwhelmingly middle class and successful, when there are bigger issues to focus on? His response magnifies why racial maneuvering is alluring: in ranking Asian American work issues as relatively minor, Jack can shift focus to “real” obstacles affecting other racial groups. Racial maneuvering makes it possible to ignore that Asian Americans’ occupational paths have historically been determined by their social standing as an “inferior race” and their social placement as an unassimilable, socially distant group (Espiritu 2008). Reinforcing model minority stereotypes enables individuals to set up Asian Americans as desirable workers but also continues to divide Asian Americans from other racial groups and minimizes their own experiences as racial subjects.

### Racial Strategy: Essentializing

Essentializing is a racial strategy that relies on popular racial stereotypes of Asian Americans as a way to explain career outcomes. Whereas racial maneuvering capitalizes on positive aspects of popular stereotypes to differentiate Asian Americans as highly desirable workers, essentializing focuses on using racial stereotypes to rationalize and accept career limitations of Asian American tech workers. Both racial strategies, however, use stereotypes about Asian Americans to make sense of career outcomes and consider the stereotypes as genuine aspects of the Asian American racial identity. Essentializers are aware of racial differences but do not acknowledge these differences as the consequence of racial inequalities. Instead, their reliance on racial stereotypes to explain the dearth of Asian Americans in leadership roles means that they understand career limitations as a function of individual choice rather than external forces.

Gary, a Bay Area-based software engineering manager, explained that he was a “good” kid that followed his parents’ advice in getting a stable, well-paying job. From his perspective, it’s “easy” to explain why the bamboo ceiling exists. First, Asian immigrant tech workers often isolate themselves in cliques and speak in their native languages, which creates a ceiling for them because they don’t expand their social network. As a manager, Gary would tell his teammates to use English in public settings and to include others in their conversations.

Second, Gary described his own upbringing as one typical of the second generation Asian American experience, where “tiger parents” instill a “do as you’re told” mentality to their offspring. His own journey into software engineering was encouraged by his parents who wanted him to pursue a “safe” (i.e. well-paying) job. Because the primary objective to enter software engineering was driven by practical needs, Gary is able to rationalize why more second generation Asian Americans do not rise in the ranks as compared to their white peers:

White people have more of a take life by the horns and follow your passion [mentality.] Those are the people who normally rise to the top or found a company. You love your job so much that you devote your life to it. Very few people are that passionate about coding…. There’s fewer Caucasian people that go into coding in the lower levels but a lot of them are truly passionate and good at it and they can rise to the top or start from the top when they start their own companies.

Gary intentionally distinguishes himself from his white peers by removing passion as an input in his career choice. His rationalization for the bamboo ceiling hinges on zeal as an explanatory mechanism for success in the tech industry. According to Gary, white peers succeed because of passion while his Asian American colleagues culturally value hard work but lack the passion it takes to pursue executive leadership positions.

And yet—despite reassuring me that he lacks passion for his field and does not “code as a hobby” in his spare time, Gary has elected to pursue a master’s degree “for fun” and move from an individual contributor role to a manager in order to improve team morale and make positive organizational change. And although not even Gary himself fits neatly into his explanation for the stilted success of Asian Americans in leadership roles, given his own drive to improve his work environment and his enjoyment of his computer science coursework, it is nevertheless a framework for Asian Americans like Gary to make sense of their world without needing to deeply examine what it means to be a racialized worker or to consider the plurality of the Asian American experience.

Other Asian Americans leaned into stereotypes of a pan-Asian culture to describe why they were not interested in moving into leadership roles. Jessica, an Austin-based product manager with over twenty years of experience in tech, shared that she had never thought about becoming part of a leadership team at work:

Our [Asian American] style of work is much more about… achieving the objective they’ve given you. Your job is not to create a space for you to move up. Your job is to get the work done, which is different from rising in the ranks. Honestly, I never thought about rising in the ranks, ever.

What Jessica describes as the Asian American style of work aligns closely with what is expected of Model Minorities: doing the work for others without promotions, although she has another term for it: Confucianism.

I think culturally we’re taught to stay low [in] a certain place. Do your work, keep your head down, be good at it, be invisible, right? In fact, isn’t it Confucian teaching, it’s very much about humility, don’t be the person who’s patting yourself on the back.

Although the core of Confucianism is about morality and social relationships, Jessica ascribes the idea of invisibility to Confucian philosophy and Asian American culture because she is not intimately familiar with Confucianism nor was she raised with its belief system. While it is a popular explanation for Asian American behaviors and outcomes (see: Lee and Zhou 2015; Xin 1997), Asian Americans like Jessica are not practicing Confucians. It is more likely that the lack of a strong collective memory of racism drives stereotypical narratives to the foreground of cultural expectations. Jessica understands it to be an Asian American ideology to stay in “a certain place” at work rather than a culture that views Asians as an economic threat and therefore teaches them to “stay low.”

Later on, Jessica reflects more deeply on her own assessment of Asian cultural values: “My perception is so skewed because I don’t question the fact that there are no Asians at the C-Suite level. I see the same thing and so it must be the same thing.” Over the course of our interview, Jessica wrestled with how her racial strategy in essentializing has affected her career expectations:

There’s a status quo that’s kind of been accepted in our minds. You just sort of accept that’s the way it is. So I think I have been discriminated against at work…. And I probably didn’t take that as [a result of my] being Asian but probably if I’m being totally honest, I’m sure there was an expectation to my level of tone and approach to things. I was expected to be a certain way… I probably made myself feel okay with [racial discrimination].

Jessica’s acknowledgement is vulnerable and demonstrates how racial strategies are used as coping mechanisms to “feel okay” with racism. Jessica’s approach requires her to accept what she sees: few Asians in leadership positions and behaving in line with what others expect.

Essentialization places the blame on Asian American career stagnation squarely on Asian Americans rather than oppressive power structures. For Gary and Jessica, essentialization provides a tidy way to attribute the lack of Asian Americans in executive roles to individual choices and ethnic cultural values but cannot fully explain their lived experiences. Ultimately, essentialization does not account for Gary’s career path given his obvious interest in computer science and his job, nor is it a fully satisfying explanation for Jessica as she continues to reflect on her work experiences.

### Racial Strategy: Distancing

Distancing was a particularly popular racial strategy because individuals arrived at it from a variety of perspectives to maintain that they did not personally experience racism in the workplace, even if other Asian Americans did. Most participants who used distancing as a strategy were aware of racial stereotypes and recognized career limitations for Asian Americans as the result of racial inequality (“bamboo ceiling”) but could not reflect on their own career trajectories using a racialized lens. Instead, distancers were satisfied with their careers and believed they had not experienced the racism that afflicts other Asian Americans. Two common ways of distancing that are examined in this section are: attributing anti-Asian racism as a “future problem,” and normalizing the Asian racial identity to the point of becoming a racially disengaged subject in the workplace.

#### Racism as a “Future Problem”

Daniel, a Bay Area-based software engineering manager originally from the East Coast, was open about his seemingly conflicting perspectives on racial discrimination in the workplace. Like other Asian American tech workers who have spent significant time thinking about racial inequality in the high-tech workforce and still strategize to minimize the effects of racism in their own lives, Daniel both acknowledges racism *in general* and believes it is “irrelevant” to his specific professional experience. In Daniel’s case, this is because he has not yet felt a barrier to promotion and because he believes he has set successful mechanisms in place to protect him from potential racist interactions.

With over two decades of experience in tech, Daniel has worked at startups as well as established tech organizations and over time has become more intentional in being an ally to women and racial minorities at work. He recognizes that racism and sexism exist in the workplace and has reflected on its impact on tech workers of color: “Like other minorities, Asians have a curse…you can succeed up to the point that the power structure allows you to and then you’re stuck after that.”

Daniel believes that Asian Americans share at least some of the disadvantages other racial groups face in the workforce and that they exist in a power structure not meant for them. However, Daniel is also able to compartmentalize this understanding from his own experience, where he feels supported by his managers and still sees potential for upward mobility: “Locally, I feel fine… I feel like I’m seeing the success that I want… playing by the rules and sort of following that at face value [in terms of] the way you get promoted and recognized has worked for me [so far].” In part, Daniel’s ability to acknowledge the racism he knows exists more broadly and separate it from his own “local” experience enables him to create the distance he needs to feel mostly unaffected by artificial barriers for Asian Americans in the workplace. And despite his reassurances that he is supported by his management team, he also reveals that he “plays by the rules” in order to ascend the corporate ladder. What exactly are those rules?

For Daniel, it means displaying certain behaviors that make him palatable as a leader to senior executives: “If I’m with some of the more senior people I will consciously be more assertive and more aggressive… I feel that I do what I can to go against type.” That Daniel modifies his behavior to preemptively counter potential stereotyping by senior leadership may seem contradictory to his assertion that he is not affected by the bamboo ceiling but Daniel sees his approach as one that “solves” for racism before it can affect him and therefore never becomes a problem. In fact, Daniel believes his approach is so successful that he shares,

I don’t really perceive any impediments [in being promoted] to the next level, to grow. I’ll be curious to see what happens if I ever try to make the jump to become a director….Let me say it more directly: the bamboo ceiling is irrelevant [to me] because I’m not close enough to it yet...There’s a couple of levels to go before it becomes a problem.

Daniel’s daily experiences at work are protected, at least for now, from the larger racial structure and empower him to dissociate his own standing from the inevitable glass barrier he will face. There are constraints to Daniel’s racial strategy—his indication that he is currently protected by his current leveling as a manager is an admission that he may well face barriers to promotion if he pursues a directorship.

This was not an unusual experience: many participants reported frustration at their homogeneous, white male led C-suite and reporting chains, while also reporting that their immediate teams provided a positive and even diverse working environment where they were not blocked from the next promotion level. Participants who used distancing compartmentalized known racial discrimination by ignoring future problems by focusing on incremental promotions within their immediate teams. Many Asian American interview participants identified the bamboo ceiling as occurring after the director level of their organization. Although Daniel is only a few levels away from a directorship at his large tech organization, the power of assigning racism as a future problem is alluring, even when the ceiling is so clearly within reach. Daniel’s experience is also indicative that racial strategies may be mutable. As Daniel advances or perhaps is unable to advance further in his career and the bamboo ceiling becomes relevant to him, he may use other racial strategies to make sense of his career trajectory.

#### Racial Privilege as a Distancing Method

Because Asian Americans who were native to the Bay Area considered themselves part of mainstream culture, I found that my Bay Area-based respondents rarely acknowledged their race as having an impact on their personal or professional lives. In fact, Bay Area Asian American tech workers often intentionally disengaged from exploring race as a potential discriminatory mechanism because they did not consider themselves marginalized or oppressed despite known racialized limitations for their careers. It was often difficult to discuss race and racism in detail with Bay Area natives because they simply had not thought about being Asian American as a marker of difference.

Sharon, a product designer with over 12 years of product design experience who has spent her entire life in the Bay Area, shared: “I don’t think about [being Asian] that much personally because where I grew up, being Asian is pretty normal.” Because Sharon grew up with a significant Asian community with others from “exactly the same ethnic background,” she was never “othered” or made to feel that her Chinese American upbringing was out of the ordinary.

Asha, another Bay Area native, shared that her family’s move to the tech suburbs was in part because her father’s friends from university had all moved to South Bay suburbia. As a result, Asha grew up with what she considers an extended family of Indian Americans, some of whom she also attended her local public school with. What she did inside her home, which included watching Bollywood movies and participating in Indian dance competitions, were interests that could be freely shared with school friends, who either participated in similar ethnic activities or were aware of their popularity.

Racial privilege for Bay Area Asian Americans like Sharon and Asha was not that their ethnic identities became optional and that they considered themselves white, but that their ethno-racial identities were socially accepted as part of mainstream culture and that they could be Asian American both at home and in public. This racial privilege, however, made it difficult for Bay Area natives to think about themselves as racial subjects because they felt socially accepted, at least within the confines of Silicon Valley. Bay Area Asian Americans rarely felt the need to confront their worldview on race and racism. As one participant succinctly summed up: “I don’t think I ever had to sit down like oh man, I’m Asian. What does it mean for me and my life, right?… I never had to sit down and ponder my Asianness.”

Whereas participants like Sharon and Asha grew up with racial privilege, Asian American transplants to Silicon Valley learned and often adapted to the racial frameworks of their peers. Asian American transplants were genuinely grateful that they were finally socially visible and that their cultural needs were so widely reflected and easily accessible. These newly minted Bay Area Asian Americans often described their move to California as a sort of homecoming: for the first time, they were witnessing Asian Americans as “insiders” and felt a sense of social belonging. John, who was raised in the suburbs of New England, shared that being in California had been personally transformative:

I just feel at home here in a way that I don’t elsewhere…. I never saw myself as the main character… if I grew up in the Bay Area, maybe I would be more inclined to see myself as the main character: ‘I’m Batman. I’m Superman.’ But I just never saw myself like that.

Whereas John previously saw himself as a secondary character, the possibility of an Asian American protagonist only came into focus after his move. John, like other migrants to Silicon Valley, relied on Bay Area natives like Asha and Sharon to recalibrate their understanding of their Asian American identity. Adopting local attitudes on race, however, also meant adopting local blind spots.

Whereas John once felt othered by white peers in his suburban upbringing and vacillated between his desire to find his place between a binary racial set up: “mainstream and white” or “hip hop and Black” he now has the privilege of being an observer in a culture where his racial identity has already been established and defined as part of mainstream culture and accepted in the workplace. John describes meetings where all of the participants are Asian and a hiring process where there is significant Asian representation. For him, this is enough to generally accept the status quo that work is not a political landscape: “It seems like the workplace is a de-politicized space. Where for whatever reason, it’s just like social norms [makes it] weird to interject that [into the conversation] even though our lives are just always entangled in it.”

Despite acknowledging that he is in fact “entangled” into politics, John is willing and able to accept this depoliticization. His conversations on race are limited to a small number of close friends mostly outside of work, and he does not participate in internal efforts on racial equality in the workforce or feel a need to discuss it with colleagues. Although John’s understanding is that Asians are generally hired to “work hard for the company [but not] run the company” he shares that he is unbothered by this since he does not see himself climbing the corporate ladder. It’s unclear whether a lifetime of being treated as a secondary character has dulled his professional expectations or whether work is simply not where he finds fulfillment but there is a lack of commitment with his thoughts on the depoliticized workplace and inertia to finding out more, as if a more complex understanding of racial inequality would change his perspective.

Although this approach seems avoidant, it is also understandable. Nearly all Asian American transplants to California struggled with their racial identity previous to the move and could describe painful racist interactions with classmates and authority figures. Their newly minted social acceptance was a relief: for the first time, they saw others who did not consider their racial identity “a handicap, or the thing that held them back from being who they were.” John, and others like him, were willing to take their social acceptance at face value and distance themselves from looming racism because they felt so much more at ease in their Asian American identity post-move.

Outside of living and working in the Bay Area, another way Asian Americans developed racial privilege was in job functions with a large proportion of Asian Americans workers and seeing them promoted into people management positions. Although this happened infrequently in tech jobs across locations because Asian tech workers are less likely to be found in leadership roles compared with their white peers (Gee and Peck 2018), Asian Americans software engineers were more likely to describe having been managed by other Asians and seeing them in technical leadership roles (e.g. Engineering Manager). This often helped tech workers choose distancing as a racial strategy because there is evidence of some Asian success within engineering departments.

Jocelyn, an early career software engineer based in the Bay Area shared that it was difficult for her to recognize and label incidents as being racially motivated unless others pointed it out because seeing the success of other Asian women in tech makes it hard to pinpoint discrimination against Asian Americans:

There are a lot of Asian women in tech and I definitely see people who are doing really well and if they’re able to do well [and] this is not totally logically thinking but the reason that you put in your head is if they are able to do really well, then it must not be a matter of race. Obviously that is not true but that’s the way people are thinking.

Although Jocelyn notes early on in our conversation that she observes the exploitation of Asian Americans by companies as individual contributors and middle management, she admits that seeing others who share her identities along gender and racial lines achieve success masks the discrimination she may face. The effect of being able to identify Asian tech workers in leadership roles, even if only within middle management or within immediate teams as a tech lead, is so powerful that Jocelyn shares that she finds it difficult to bring race as a framing device into her own professional experience.

For example, it wasn’t until Jocelyn replayed a conversation she had at work with her parents, who suggested that the feedback she received might have been racially motivated, that Jocelyn considered how race might affect her at work. Jocelyn acknowledges that while others may be more sensitive to this connection, she herself does not think in those terms because the visible success of other Asians makes it difficult for her to center negative experiences around her racial identity.

For Asian Americans who are fortunate enough to see others like them succeed along a career path that they consider successful, distancing as a racial strategy makes sense because there is evidence that someone like them has been allowed to succeed. Asian visibility, often described as “overrepresentation,” is used to suggest that Asian tech workers are not discriminated against in the tech industry, at least in its most well-known expression of racism, barrier to entry, despite evidence to the contrary (Chavez 2020; Petersen et al. 2000). The specific form of racism around career mobility Asian Americans are more theoretically familiar with is seemingly often less obvious and less acutely felt in their daily lives, especially for engineering departments where Asian managers are anecdotally more common. Asian American visibility in tech suggests that race is not a factor in career outcomes and makes it possible for Asian Americans to distance themselves from the burden of considering their racial identity as a factor in achieving professional milestones.

### Racial Strategy: Dissenting

Although the previous three strategies focused on separating Asian Americans from race and racism, a fourth racial strategy intentionally engages with anti-Asian discrimination in the workplace. This strategy frequently had a spatial component: Bay Area Asian Americans often had to leave California to recognize their racial privilege and Asian Americans who grew up in non-Asian majority communities had more encounters with blatant racial discrimination that made it more difficult for them to ignore the realities of being a racial minority. Those who dissented as a racial strategy refused to accommodate racialized stereotypes of Asian Americans and often left mainstream organizations in order to feel in control of their careers. In this section, dissenters share their frustration with peers and organizations that refuse to acknowledge their racial biases and divulge how they cope with racial disadvantage in the high-tech industry.

For Bay Area Asian Americans, leaving California was often a turning point in developing their racial consciousness. Chris, a Bay Area native, shared that his time in the Midwest transformed his understanding of race: “I used to travel around the Midwest for [university] and I would get stared at when we were at rest stops because I’m an Asian person that people don’t really see at [these] stops. People [from the Bay Area] don’t have these experiences and see themselves as the white people of the Bay Area.” Chris’ experiences in the Midwest removed him from an environment where he had only been part of a majority and helped him understand that Asian Americans faced “othering.” After his return to the Bay Area, Chris was able to more critically reflect how Asian Americans’ racial privilege prevented them from acknowledging any possibility of anti-Asian discrimination in his hometown. Chris points to the number of Trump supporters among his friends’ parents and their rallying behind his rhetoric: “They see other racial minorities as the others.”

Asian Americans who grew up more racially isolated were forced to reckon with their racial identity earlier in their personal lives. Although some transplants to the Bay Area, such as John and Jocelyn, found freedom in being Asian American in their adopted state and flourished under their newfound racial privilege, not all were swayed by its allure. Divye, a product manager who now freelances in the Pacific Northwest but moved to Silicon Valley in the late aughts and spent nearly a decade there, recalled his Asian American and white friends dismissing racism something as “not something that happens [in the Bay Area]” because they had all gone to school together and believed it to be an integrated environment. To them, Divye was overly sensitive on issues of race and his experiences of racism in the Midwest were “backwards” and “coming from another age” whereas Silicon Valley represented an integrated present.

Because Divye could recall specific and frequent racist incidents in his childhood and in his professional experiences working in the Midwest, he was particularly observant of race coming into the Bay Area. As he pointed out to his friends: “Who’s getting the accolades? Who gets the awards? Who gets the VC money that floats around Silicon Valley? You [Asian Americans] are still not represented. They [white people] may make you feel like you’ve been represented just because they’re acknowledging your presence.”

Dissenters like Divye were not satisfied with simply being represented in tech; instead, they keenly observed and protested the racial inequality Asian Americans faced in the industry, from venture capital dollars to awards to promotions. Knowing that he could not “trust” an organization to develop his career in a way that recognized his potential, Divye turned to freelancing in order to take control of his career despite the fact that this path requires more effort and often creates additional stress given the instability of contract work.

In Austin, Asian Americans also rallied against stereotypes. Jin, who was raised in the Deep South, noted that her reserved working style was racialized and prevented her from being promoted into leadership positions. She routinely received feedback that she needed to be more “charismatic,” which she recognized would not be the case if she were a white man, in which case Jin speculated her humble and hardworking nature would be rewarded.

Despite having led multiple successful projects at a previous company, she was told that she did not have a personal “brand” for leadership to understand her value: “[As an Asian American] you are a good worker horse but you’re going to have to be fucking Charlie Chaplin if [white leaders] are going to [promote] you. You have to perform much more.”

As Jin’s analysis points out, it wasn’t enough for her to do the work: she would also have the additional burden of refuting racial stereotypes in order to be accepted as part of the leadership team. Because Jin was unwilling to “perform” for their acceptance, she was repeatedly told she was “not ready” to move into a directorship despite managing large projects that others deemed “impossible.” Eventually, the frequency in which she was questioned whether she could sell projects to clients caused her to intentionally disengage because it was too hurtful to be passed over repeatedly. Like Divye, Jin left mainstream organizations: she first freelanced and then started her own company: “I’ve felt overwhelmingly that I had to leave that system [the white mainstream organization] in order to achieve my own potential. It felt like a ceiling, a very clear one.”

Both Jin and Divye struggled with the consequences with their dissent from the status quo. For Divye, it was difficult relating to the larger racial structure of the Bay Area. He was unable to genuinely connect with other Asian Americans on race. Later in his career, he moved to Austin where he faced workplace racism that prevented him from effectively been seen as a team player and being favorably evaluated during annual reviews. Jin’s refusal to “turn on” her charm prevented her move into leadership. Dissenting as a racial strategy is clear on racism against Asian Americans but is misaligned with the cultural realities of the tech industry and can drive Asian Americans out of the tech industry.

## Conclusion

This article reframes the notion that Asian Americans are simply white-adjacent subjects and receive white-adjacent privileges in tech. I introduce four racial strategies Asian Americans use to interpret racialized experiences in the workforce: racial maneuvering, essentializing, distancing, and dissenting. The first two strategies demonstrate the ways in which many Asian Americans have internalized racial stereotypes of Asian Americans and adopted racial privileges generally available only to white Americans; the other two racial strategies—distancing and dissenting—recognize racial inequality for Asian Americans in the workplace but differ in understanding its impact. Among the racial strategies available, only those who use the dissenting strategy choose to be active agents in resisting white power structures in the workplace. By introducing the concept of racial strategies, I situate Asian Americans as racialized subjects who are affected by workplace racism and react to it in complicated ways in order to survive professionally.

Although this research project is specifically about a select group of white-collar Asian American tech workers, it demonstrates the complexity of the Asian American occupational experience and the importance of local environments. As established through the stories of Asian Americans from across the country, their personal histories deeply shape how they perceive racism in other facets of their lives. In general, those who have never had to deal with racism find it particularly hard to recognize racial inequality in the workplace whereas those who have been racially isolated from Asian American communities are more likely to be aware of racism in both their personal and professional lives. The Bay Area, and more broadly, Asian majority-minority communities in California, serves as a protective racial shield for Asian Americans. Because Asian American tech workers see themselves as part of the mainstream culture in Silicon Valley, this privilege often blinds them from being able to recognize racial discrimination levied at Asian Americans. As a result, Asian Americans working in the Bay Area often lack the ability to converse about race as it relates to the Asian American experience meaningfully.

Several events and ideological shifts have occurred since I finished interviewing participants that may shift the way Asian American tech workers understand their racial identity: first, the high tech industry has shifted to a more remote work environment in response to COVID-19, marking potentially new means of establishing racial hierarchies in the virtual workplace and potentially amplifying—or lessening—the scope of Bay Area racial privilege in the tech workforce; and two, visibility into anti-Asian violence and increasing awareness of Asian Americans as racial subjects has the potential to activate Asian Americans in the efforts toward racial justice. The latter ideological shift comes on the heels of the pandemic, which associated the coronavirus with China and East Asians regardless of nationality, and the Black Lives Matter Movement, which gained wider public support in 2020 and galvanized many of the participants in this study to think more closely about race and their participation in white supremacy.

These changes demonstrate how quickly racial ideologies change and adapt to world events. As such, my interviews capture a particular moment in time: when tech work was still mainly a spatially-based industry that agglomerated in specific geographic regions (Moretti 2013; Saxenian 200), and before the re-emergence of “Yellow Peril” stereotypes which are now more frequently and violently in the open (Tessler et al. 2020). Future scholars have the opportunity to focus on the evolving understanding of racial identity among Asian Americans in the workplace and their relationships with other racial minorities in the tech industry. Possible lines of research could address whether Asian American racial strategies differ in less Asian-concentrated industries and whether we would expect Asian Americans in other locales to strategize similarly. Given the recent catalysts for organizing around Asian American specific racial projects, there is hope that Asian American tech workers will meaningfully contribute to larger racial justice initiatives and the communities around them.
